# Prospective study of the efficacy of antibiotics versus antitussive drugs for the management of URTI-related acute cough in children

**DOI:** 10.1186/s40248-016-0059-y

**Published:** 2016-06-13

**Authors:** Alessandro Zanasi, Luigi Lanata, Federico Saibene, Giovanni Fontana, Peter V. Dicpinigaitis, Valentina Venier, Francesco De Blasio

**Affiliations:** Pneumology Unit, University of Bologna, S.Orsola Malpighi Hospital, Bologna, Italy; Medical Department Dompè Farmaceutici SpA, Milan, Italy; University Hospital Careggi, Florence, Italy; Department of Medicine, Albert Einstein College of Medicine and Montefiore Medical Center, Bronx, NY USA; Respiratory Medicine and Pulmonary Rehabilitation Section, Clinic Center, Private Hospital, Naples, Italy

**Keywords:** Levodropropizine, Cough, Antibiotic, Antitussives, URTI, Children

## Abstract

**Background:**

Acute cough is one of the most frequent symptoms prompting a visit to a health care provider, usually following a viral upper respiratory tract infection (URTI). The disproportionate use of antibiotics in children with URTIs, recently highlighted in the medical literature, could lead to associated side effects, without any beneficial effect. Although an early, albeit inappropriate, antibiotic prescription increases parental satisfaction, URTIs are predominantly viral infections and are generally self-limiting. Therefore the aim of this study was to analyze the effectiveness of antibiotics compared to symptomatic drugs (central and peripheral antitussives) on URTI-related cough in a pediatric population.

**Methods:**

This is a prospective observational study of 330 children who required pediatric consultation for acute cough. Severity, frequency and type of cough were assessed at baseline and after 6 days of treatment (antitussives *n* = 123, antibiotics *n* = 89 or combination of them *n* = 38) or no treatment (*n* = 80). The outcome of cough management after 6 days was analyzed in terms of resolution, improvement, no change or worsening of symptoms. Study assessments were performed using a standardized questionnaire administered to parents.

**Results:**

Between children treated with antitussives or antibiotics, there was a statistically significant difference in the resolution of cough. Moreover, if considering peripheral antitussives, the resolution of cough was significantly higher with antitussives than with antibiotics (*p* < 0.01). There was no difference in cough resolution between children treated with antitussives and those receiving a combination of antibiotics and antitussives, either central and peripheral antitussives.

**Conclusion:**

Antibiotics are generally not useful nor appropriate in treating acute cough due to the common cold. Furthermore, inappropriate antibiotic use introduces the possibility of adverse side effects as well as promotion of antibiotic resistance. The findings of the present study suggest that antitussives, especially peripherally acting agents, represent an effective treatment option for acute pediatric cough caused by URTIs.

## Background

Antibiotic resistance of pathogenic bacteria is being recognized as a major emerging threat in healthcare settings throughout the world. In addition, the discovery of new molecules with antimicrobial activity is no longer keeping pace with the spread of resistant bacterial pathogens. Therefore local and international efforts and strategies are needed to neutralize this emerging threat [1]. The main contributor to the development, increase and spread of antibiotic resistance is the overuse of antibiotics, particularly in children. Of particular concern is the over-prescription of these drugs to children for the treatment of upper respiratory infections (URTIs) and influenza-like illness.

URTI, the most commonly treated acute problem in primary care, is a pathological condition most often caused by viruses [2, 3] and thus, does not require antibiotics [4−6]. However, URTIs are the conditions for which the misuse of antibiotics is particularly high and reported worldwide [7, 8] despite the strong evidence on their self-limiting nature. Even some of the bacterial illnesses (such as otitis media and sinusitis) are usually self-limited and antibiotic treatment is not recommended for these conditions [9].

An important factor leading to the inappropriate use of antibiotics in children with URTIs is the difficulty in making a reliable and rapid clinical diagnosis. In fact, distinguishing between the clinical features of influenza and bacterial infections is the main challenge for physicians. Furthermore, doctors may prescribe antibiotics as a response to parents’ expectations. A survey of more than 600 pediatricians showed that 96 % had been asked by parents for antibiotics in circumstances for which they were unnecessary [10].

URTI or common cold are by far the most common cause of acute cough, defined as cough of recent onset and lasting for a maximum of 3 weeks. For the treatment of acute cough, symptomatic over-the-counter (OTC) drugs, such as antitussive or mucoactive drugs, are frequently recommended as a first-line intervention [11].

Mainly, two classes of antitussive agents are available for the treatment of cough in children: centrally acting cough suppressants (codeine, dextromethorphan and cloperastine), and peripherally acting antitussives such as levodropropizine, a non-opioid inhibitor of the cough reflex at the peripheral nerve level (sensory C fibres) and a modulator of sensory neuropeptides within the respiratory tract [12]. Hence, in order to analyze the effectiveness of antibiotics compared to symptomatic drugs in improving or eliminating cough caused by URTI, we carried out an analysis of data collected from a prospective study comparing children, with URTI and cough, treated with antitussive drug as symptomatic therapy or antibiotics or their combination.

## Methods

This study is an analysis of data collected during a prospective observational study performed in 2012 [13]. It was performed in children who required a pediatric consultation for acute cough of recent onset (≤3 weeks) caused by URTI, from 1st February to 30th April 2010.

Study assessments were performed through a standardized Pediatric Cough Questionnaire (PCQ) developed and approved by the Scientific Committee of the Italian Society of Cough Study. The PCQ consists of two different parts.

Baseline assessment regarding type (dry, productive, mix), frequency, duration and severity of cough (defined as: mild if it did not interfere with common daily activities, moderate if occasionally disturbed common daily activities and severe if interfered with daily activities and night rest) was performed during the first study visit by the pediatrician, who interviewed parents and/or patients, compiled the first part of the PCQ and prescribed the most appropriate treatment for cough.

The PCQ was given to the children’s parents to complete the second part 6 days after the first study visit, to document administered treatments and to self-assess outcome of therapy in terms of resolution (cough disappearance), improvement (just few sporadic cough spells), no change in symptoms or worsening of cough (more frequent and severe cough).

Patients presented 1 week after the first visit at which time questionnaires were collected by the physician. Any adverse events were also reported.

According to the aim of this study, questionnaires of patients reported specific types of treatment; in particular, antibiotics or antitussives, combination of antibiotics and antitussives, and no pharmacological treatment, have been selected for analysis.

### Statistics

Continuous variables are presented as mean +/− standard deviation. Categorical and discrete variables are presented as frequency and percentage. Differences between groups were tested using χ^2^ test for categorical and discrete variables (with Yates correction for 2x2 tables). Analysis of variance (ANOVA) has been used to analyze the differences between groups for the following two parameters: age (expressed as years) and duration of cough (expressed as days).

The correlation between the treatment and the type of cough (productive, mix or dry), the episodes of cough (occasional, frequent or continuous) and the severity of cough (mild, moderate, severe) was performed by the polychotomous stepwise logistic regression.

## Results

### Epidemiology of cough and clinical findings

In total, 330 children affected by URTI were evaluated in this analysis. Clinical data including frequency, severity and type of cough were recorded on the PCQ during the first study visit. Tables 1 and 2 reported patients’ characteristics at baseline according to different types of treatments.

**Table 1:** Patients characteritics at baseline according to types of treatments

	Antibiotic (ANT) *n* = 89	Antitussives (ATT) *n* = 123	Combination (COM) *n* = 38	No Treatment (NT) *n* = 55	*p*
Age (years), mean (min-max)	4.9 (0.4–13.0)	6.4 (0.8–14.0)	6.5 (1.7–13.3)	7.0 (0.3–14.0)	*p* < 0.01
Cough Duration (days), mean (min-max)	5.7 (1.0–21.0)	4.6 (2.0–20.0)	6.5 (2.0–30.0)	5.0 (2.0–1.0)	*p* < 0.05
Cough Type (%)					
Productive	61.8 %	26 %	52.6 %	47.3 %	*p* < 0.01
Dry	23.6 %	64.2 %	26.3 %	34.5 %	
Mix	14.6 %	9.8 %	21.1 %	18.2 %	NS ANT vs ATT, NS ATT vs COM
Cough Intensity (%)					
Mild	19.1 %	10.6 %	0 %	43.6 %	NS ANT vs ATT
Moderate	36 %	63.4 %	34.2 %	54.5 %	NS ANT vs ATT, NS ATT vs COM
Severe	44.9 %	26 %	65.8 %	1.8 %	NS ANT vs ATT
Cough Frequency (%)					
Occasional	22.5 %	20.3 %	0 %	58.2 %	NS ANT vs ATT
Frequent	58.4 %	71.5 %	60.5 %	41.8 %	NS ANT vs ATT, NS ATT vs COM
Continuous	19.1 %	8.1 %	39.5 %	0 %	NS ANT vs ATT
Concomitant respiratory diseases					
Yes	74.2 %	72.4 %	81.6 %	92.7 %	*p* < 0.05
No	25.8 %	27.6 %	18.4 %	7.3 %	
First episode of cough					
Yes	57.3 %	51.2 %	63.2 %	40 %	NS
No	42.7 %	48.8 %	36.8 %	60 %	NS

**Table 2:** Cough characteristics at baseline according to types of administered antitussives (centrals vs levodropropizine)

	Central Antitussives (CA) *n* = 44	Levodropropizine (LDP) *n* = 79	*p*
Cough Type (%)			
Productive	25 %	75 %	0,1773 (NS)
Dry	41.8 %	58.2 %	
Mix	25 %	75 %%	
Cough Intensity (%)			
Mild	38.5 %	61.5 %	0.7517 (NS)
Moderate	33.3 %	66.7 %	
Severe	40.6 %	59.4 %	
Cough Frequency (%)			
Occasional	24 %	76 %	0.3869 (NS)
Frequent	38.6 %	61.4 %	
Continuous	40 %	60 %	

### Treatment of cough

**Fig. 1:**
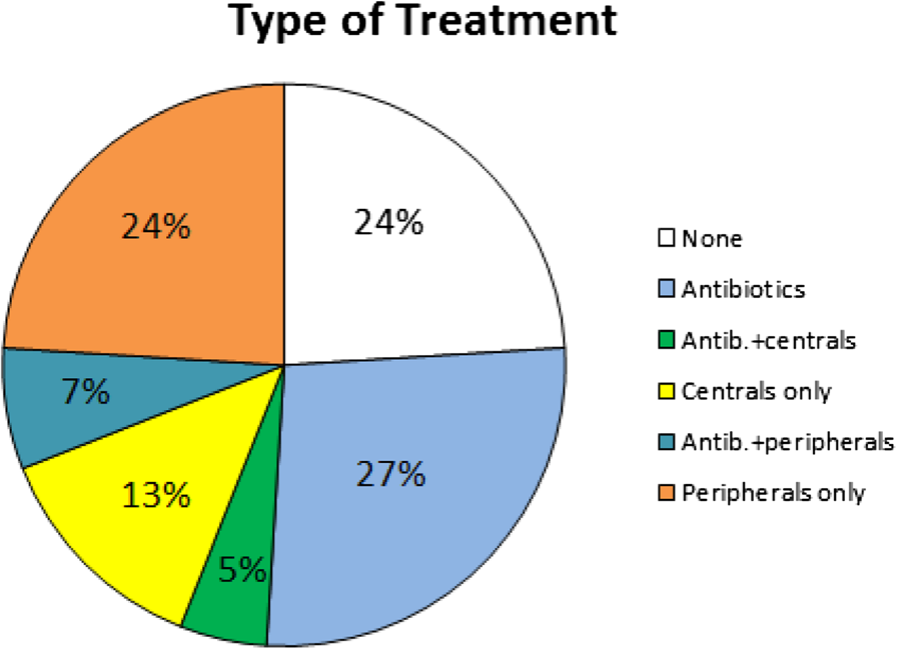
Types of treatments administered to children included in the study

Eighty-nine children (27 %) received antibiotics, while 38 (12 %) children received a combination of antibiotics and antitussives; central antitussives (codeine or cloperastine) were given in 16 cases (5 %), and peripheral antitussives (levodropropizine) were given to 22 children (7 %). Forty-four and 79 children received central or peripheral antitussives respectively (13 and 24 %), without antibiotics. Eighty children (24 %) did not receive any treatment for cough (Fig. 1).

### Correlation between treatments outcomes and type of treatment received

**Fig. 2:**
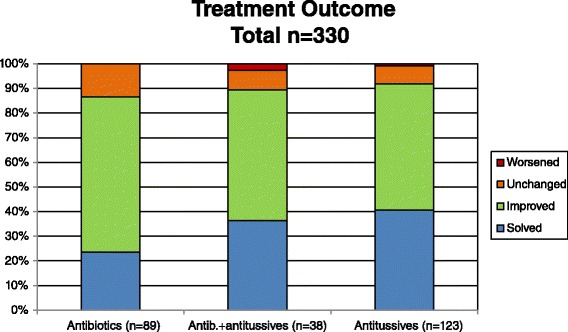
Treatments outcomes according to different types of administered treatment

Fifty-one per cent of patients treated with antitussives had an improvement in cough, 41 % reported resolution of cough, and 7 % did not have any change (Fig. 2).

**Fig. 3:**
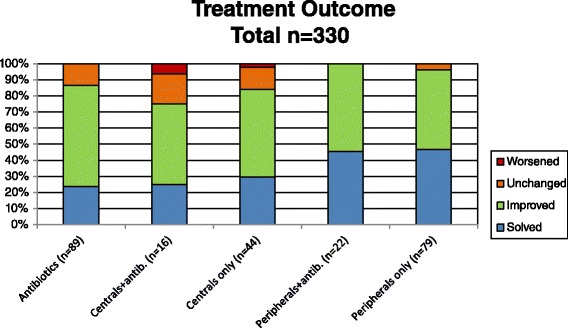
Treatments outcomes according to different types of treatment including the distinction between central and peripheral antitussive molecules

Among the patients receiving peripheral antitussives (levodropropizine), 49 % reported improvement in cough, and 47 % reported the resolution of cough. Four per cent did not have any change in cough symptoms. In the group of children receiving central antitussives, 54.5 % reported improvement in cough, 29.5 % reported resolution of cough, 14 % did not have any change in symptoms and 6 % reported worsened symptoms (Fig. 3).

Fifty-three per cent of patients treated with both antibiotics and antitussives reported improvement in their symptoms. The resolution of cough was reported in 37 % of children. Eight per cent of patients did not have any change and 3 % reported worsening of their condition (Fig. 2).

Considering the patients treated with antibiotics plus levodropropizine, 54.5 % noted improvement in their symptoms and 45 % reported resolution of cough. In the group of patients receiving both antibiotics and central antitussives, 50 % reported an improvement of symptoms, 25 % reported cough resolution, 19 % did not have any change in cough symptoms and 6 % worsened (Fig. 3).

Considering patients treated with antibiotics, 63 % reported an improvement, 24 % reported resolution of cough and 13.5 % did not have any change (Fig. 2).

There was no statistically significant difference in the resolution of cough between patients treated with antitussives and those treated with the combination of antitussives and antibiotics (χ^2^ = 0.053; *p* = NS).

Comparing children treated with antitussives or antibiotics, there was a statistically significant difference in the resolution of cough (χ^2^ = 5.99; *p* < 0.05) in favor of antitussive drugs.

Including in the analysis the distinction between central and peripheral antitussives, the difference in the resolution of cough between children treated with a peripheral agent (levodropropizine) or antibiotics alone was statistically significant (χ^2^ = 8.998; *p* < 0.01) in favor of levodropropizine. This result did not change when considering only children with severe cough at baseline (χ^2^ = 8.928; *p* < 0.01). On the contrary, the resolution of cough was not statistically different between children treated with central antitussives or antibiotics (χ^2^ = 0.280; *p* = NS).

All statistical analysis results are reported in Tables 3 and 4.

**Table 3:** Outcome differences between group of treatment: χ^2^ test results

Treatments	Resolution of cough
Antibiotics vs antitussives	χ^2^ = 5.999 (*p* < 0.05)
Antibiotics vs peripheral antitussives	χ^2^ = 8.998 (*p* < 0.01)
Antibiotics vs central antitussives	χ^2^ = 0.280 (*p* = NS)
Antibiotics + antitussives vs antitussives	χ^2^ = 0.053 (*p* = NS)
Antibiotics + peripheral antituss. vs peripheral antitussives	χ^2^ = 0.016 (*p* = NS)
Antibiotics + central antitussives vs central antitussives	χ^2^ = 0.001 (*p* = NS)

**Table 4:** Outcome differences between group of treatment in children with severe cough: χ^2^ test results

Treatments	Resolution of cough
Antibiotics vs antitussives	χ^2^ = 3.738 (*p* = 0.0532, NS)
Antibiotics vs peripheral antitussives	χ^2^ = 8.928 (*p* < 0.01)
Antibiotics vs central antitussives	χ^2^ = 0.000 (*p* = NS)
Antibiotics + antitussives vs antitussives	χ^2^ = 0.218 (*p* = NS)
Antibiotics + peripheral antituss. vs peripheral antitussives	χ^2^ = 0.000 (*p* = NS)
Antibiotics + central antitussives vs central antitussives	χ^2^ = 0.713 (*p* = NS)

*NA* not applicable

## Discussion

An interventional strategy is necessary to reduce antibiotic misuse and overuse in pediatric primary care for the treatment of acute cough, including the promotion of other beneficial approaches, such as medications for the amelioration of troublesome symptoms associated with URTI. The use of antibiotics in pediatric URTI remains controversial, despite evidence that the vast majority of URTIs has a viral origin. In addition, even if URTIs are caused by bacteria, the probability of their resolution without the administration of antibiotics is high [14]. Since cough is usually the major cause of discomfort in patients presenting with URTI, a symptomatic treatment with antitussive drugs appears to be a reasonable approach to the management of this condition [9].

Nonetheless, from the analysis of questionnaires we found that in the group of children receiving a pharmacological treatment (76 %) for their URTI symptoms, half were prescribed antibiotics with or without antitussive drugs while the remaining 38.5 % were treated with peripheral or central antitussives.

One important and clinically relevant finding of our study is that there was no statistically significant difference in the percentage of cough resolution between children treated with antitussive compared to children receiving a combination of antibiotics and antitussives. Furthermore, cough resolution in children treated with antitussives was significantly higher than in children treated with antibiotics. We also observed that after a week of treatment, levodropropizine significantly solved cough greater than antibiotics did.

Furthermore, this result did not change when considering only children with severe cough at baseline.

Taken together, these findings confirm that the use of antibiotics is of little benefit and it may be not necessary in the symptomatic management of cough associated with URTI, consistent with a previous report [15] in which the management of acute moist cough in the presence of URTI by means of antimicrobial therapy was not superior to the treatment with inhaled mucoactive drugs in the pediatric setting.

The main limitation of our study is its observational design, thus lacking treatment randomization, blinding of both patients and outcome assessors, and homogeneity in groups for treatment at baseline. However, we wanted to evaluate in a real-life pediatric setting the management of acute cough associated with URTI, comparing also the efficacy of the two main therapeutic approaches currently in use: antimicrobial vs antitussive therapy.

Finally, another possible limitation is the lack of a microbiological characterization of the URTI. Nonetheless, in the presence of URTI, guidelines for the management of cough in children do not recommend microbiological testing [15, 16] but rather, a strategy of no antibiotic or delayed antibiotic prescription, is recommended [15, 17].

## Conclusion

Our observational results suggest that acute cough associated with URTI can be effectively managed with symptomatic therapy alone, i.e., central or peripheral antitussives, yielding the same clinical benefits compared with combination therapy that included antibiotics. the present study, levodropropizine appeared to be the most effective option at relieving cough. In light of previously demonstrated efficacy data [12, 18, 19] and the considerable safety profile of levodropropizine [18, 20], these results further support its use for the management of acute pediatric cough associated with URTI. Further large randomized clinical trial in children should be conducted in order to confirm the effectiveness of antitussive drugs used in this observational study.
